# Competing for public funding of medicines to treat rare disorders in New Zealand

**DOI:** 10.2471/BLT.14.148189

**Published:** 2015-02-01

**Authors:** Steffan Crausaz

**Affiliations:** a Chief Executive, PHARMAC, PO Box 10254, Wellington, 6143, New Zealand.

Meeting the high and increasing price of medicines for rare disorders is an ongoing dilemma for patients, insurers and governments worldwide. In New Zealand, the difficulty has been highlighted by lobbyists campaigning for funding eculizumab for paroxysmal nocturnal haemoglobinuria and for enzyme replacement therapies including alglucosidase alfa for glycogen storage disease type II. The cost per patient per year for these treatments can be more than 500 000 New Zealand dollars (NZ$).

The pharmaceutical industry has long argued that its pricing of such products is justified by high development costs and the comparatively small patient population from which they can recover these costs. The validity of this argument has been recently questioned, as the cost of development is often shared or subject to tax incentives.^1^

New Zealand’s pharmaceutical management agency (PHARMAC) manages applications for funding of pharmaceuticals that are subsidised by the government for treatment in the community and dispersed free of charge for inpatients of public hospitals. Within its finite budget, the agency compares spending opportunities over several dimensions, including health needs of the population and effectiveness and cost–effectiveness of medicines. This means carefully selecting which products offer the best value and prioritizing new investments to ensure best health outcomes overall. Medicines with high prices do not usually compare favourably when decisions are made about how best health outcomes are achieved with the available funding – PHARMAC’s over-arching objective.

The agency has to consider benefits and costs as well as the opportunity cost of spending large amounts on low value-for-money products. This overall imperative exists despite the fact that some medicines for rare disorders can offer reasonable health benefits to high-need patients. One such product – eculizumab – was declined funding in November 2013.

New Zealand uses a unique approach by combining clinical and economic analysis with budget management and commercial procurement methods to decide which medicines will be subsidised. Its pharmaceutical management agency has the authority to decline funding and can negotiate with industry over price and access criteria. These methods promote competition among suppliers, who compete for the same limited pool of funding. This process has led to New Zealand having some of the lowest prices in the world for many medicines.^2^

However, due to the small number of suppliers of medicines for rare disorders, there is a lack of competition and prices remain high for these products. To address this problem the agency ear-marked up to NZ$ 5 million per year that would be available to fund medicines specifically for rare disorders. This funding came from its named patient pharmaceutical assessment policy which is used to assess applications for individual patients seeking access to medicines not funded on the pharmaceutical schedule.

With input from expert clinicians and public consultation, the agency developed a set of prerequisites for medicines that would be eligible for consideration under the rare disorders funding process. Criteria for inclusion are similar to those used for Australia’s orphan drugs policy.^3^ Such medicines need to have a proven level of efficacy, offer significant health gains for patients and the target conditions need to be rare; one case per 50 000 people.

The agency then issued a request for proposals to the pharmaceutical industry. This set out the type of products sought, the conditions to be treated and the process for making offers to PHARMAC. Submissions closed in late September 2014. The request succeeded in attracting many offers from companies seeking funding for medicines for rare disorders. The total numbers of proposals received will be made public soon. Following clinical advice and assessment, decisions and listing of products are likely to occur from early 2015. Funding for the products agreed through this process will continue indefinitely. The agency will evaluate results before issuing another request for proposals. Ultimately, this fund is about promoting competition among suppliers, an area where PHARMAC has an established track record that has enabled New Zealand to achieve some of the lowest prices for medicines in the world. 
